# Supplementary material for: Contraceptive use among sexually active women living with HIV in western Ethiopia
Source: PLoS One. 2020 Aug 6;15(8):e0237212. doi: 10.1371/journal.pone.0237212 (PMC7410321; doi:10.1371/journal.pone.0237212)
Supplement: Attachment — Submitted filename: Clean Response to reviewers 2 09 03 2020.docx [file pone.0237212.s001.docx]

4^th^ March 2020

Dear Dr Zelalem T. Haile

Thank you for considering our recent manuscript “**Contraceptive use among sexually active women living with HIV in western Ethiopia”**. We greatly appreciate the effort and comments from the reviewers, as well as from the journal editors.

In response to your letter dated January 30, 2020 regarding our manuscript, we would like to provide the following clarifications and amendments. We have attached both a clean and annotated version of the revised manuscript.

# Journal Requirements:

1. When submitting your revision, we need you to address these additional requirements.
   Please ensure that your manuscript meets PLOS ONE's style requirements, including those for file naming. The PLOS ONE style templates can be found at
   <http://www.journals.plos.org/plosone/s/file?id=wjVg/PLOSOne_formatting_sample_main_body.pdf> and <http://www.journals.plos.org/plosone/s/file?id=ba62/PLOSOne_formatting_sample_title_authors_affiliations.pdf>

Response: As per the comment, PLOS ONE's style requirements have been followed.

2. We note that you have indicated that data from this study are available upon request. PLOS only allows data to be available upon request if there are legal or ethical restrictions on sharing data publicly. For information on unacceptable data access restrictions, please see <http://journals.plos.org/plosone/s/data-availability#loc-unacceptable-data-access-restrictions>.
   In your revised cover letter, please address the following prompts:
   a) If there are ethical or legal restrictions on sharing a de-identified data set, please explain them in detail (e.g., data contain potentially identifying or sensitive patient information) and who has imposed them (e.g., an ethics committee). Please also provide contact information for a data access committee, ethics committee, or other institutional body to which data requests may be sent.

b) If there are no restrictions, please upload the minimal anonymized data set necessary to replicate your study findings as either Supporting Information files or to a stable, public repository and provide us with the relevant URLs, DOIs, or accession numbers. Please see <http://www.bmj.com/content/340/bmj.c181.long> for guidelines on how to de-identify and prepare clinical data for publication. For a list of acceptable repositories, please see <http://journals.plos.org/plosone/s/data-availability#loc-recommended-repositories>.
We will update your Data Availability statement on your behalf to reflect the information you provide.

Response: Due to the presence of potentially sensitive information provided by women living with HIV, data have been made available upon request. This requirement was imposed by Human Research Ethics Committee of The University of Newcastle, Australia, and the Oromia Regional State Health Bureau Research Ethics Committee, Ethiopia which approved the research protocol. The data request may be submitted to the Research Centre for Generational Health and Ageing, University of Newcastle, Australia at [rcgha@newcastle.edu.au](mailto:rcgha@newcastle.edu.au). This information has been amended in the cover letter as per request.

3. Please amend your current ethics statement to address the following concerns: Please explain why was written consent was not obtained, how you recorded/documented participant consent, and if the ethics committees/IRBs approved this consent procedure.

Response: Additional details regarding the ethical considerations have been added to the methods section. The section now reads:

Ethical approval for this study was obtained from The Human Research Ethics Committee (HREC) of The University of Newcastle, Australia (H-2017-0289), and the Oromia Regional State Health Bureau Research Ethics Committee, Ethiopia (BEFO/HBISH/1-16/257). Official permission was obtained from hospitals, health centers, and respective HIV-clinics included in our study. An information statement was provided to all participants prior to obtaining informed verbal consent. To ensure informed verbal consent from participants, the data collectors read the information statement in the local language, Oromo. There are low literacy levels among women in Ethiopia, (42% of women in Ethiopia are literate) (Central Statistical Agency (CSA) [Ethiopia] and ICF 2016), so informed verbal consent was more appropriate and was approved by both ethics committees. In addition, it was a survey and the research involved no more than low risk (FDRE Ministry of Science and Technolgy 2014). Participants were given the opportunity to ask questions prior to the interview. The consent procedure took place in a separate private room by female nurse data collectors after WLHIV had finished their routine clinical care appointment. Participants were informed that their participation was voluntary and that they were free to decline participation or withdraw their consent at any time. It was made clear that participation in this study had no bearing on their receipt of clinical care. The participants were also informed that the survey involved some questions that they might find embarrassing or too personal and some that might cause them to worry about their reproductive health issues. Further, participants were informed that they did not have to answer any question that they did not feel comfortable with, and they could withdraw at any time or simply choose not to answer a particular question. Female nurses were prepared to provide psychological support if the need arose. Anonymised data were stored on password-protected Ipads during data collection. Data were stored on secure and password-protected computers.

4. Please include additional information regarding the survey or questionnaire used in the study and ensure that you have provided sufficient details that others could replicate the analyses. For instance, if you developed a questionnaire as part of this study and it is not under a copyright more restrictive than CC-BY, please include a copy, in both the original language and English, as Supporting Information.  If the original language is written in non-Latin characters, for example Amharic, Chinese, or Korean, please use a file format that ensures these characters are visible.

Response: Study tools in English and Oromo languages have been adapted from the Demographic and Health Survey (<https://dhsprogram.com/pubs/pdf/FR328/FR328.pdf>) (Central Statistical Agency (CSA) [Ethiopia] and ICF 2016) and Family Planning 2020 questionnaires (<https://www.familyplanning2020.org/>) (Cahill, Sonneveldt et al. 2018). This information has been added to the manuscript. As the tools are subject to copyright they have not been included as supporting files.

5. Please state whether you validated the questionnaire prior to testing on study participants. Please provide details regarding the validation group within the methods section.

Response: Details regarding the reliability and validity of the questionnaire have been included in the manuscript. Pretesting was carried out to ensure the validity of the questions. Moreover, to ensure validity and reliability of study tools, the questions were mainly adapted from the Demographic and Health Survey (Central Statistical Agency (CSA) [Ethiopia] and ICF 2016) and Family Planning 2020 questionnaires (Cahill, Sonneveldt et al. 2018), which were widely tested in many countries, including Ethiopia. The addition to the manuscript regarding the reliability and validity of the questionnaire reads:

To ensure validity (measuring what it aims to measure) and reliability (consistency of measurements) (Heale and Twycross 2015) of the questionnaire, the questions were mainly adapted from the Demographic and Health Survey (Central Statistical Agency (CSA) [Ethiopia] and ICF 2016) and Family Planning 2020 questionnaires (Cahill, Sonneveldt et al. 2018), which were widely tested in many countries, including Ethiopia. Because of ethical and logistic reasons, a pilot study was carried out on 30 participants in the health facilities that were selected for main data collection.

6. Thank you for stating the following in the Acknowledgments Section of your manuscript:

We would like to thank study participants and data collectors for making this research successful. TRF is supported by The University of Newcastle International Postgraduate Research Scholarship (UNIPRS) and The University of Newcastle Research Scholarship Central 50:50 (UNRSC 50:50). Dr Melissa Harris is supported by an Australian Research Council Discovery Early Career Researcher Award (DECRA). We would also like to thank Natalia Soeters for language edition.

We note that you have provided funding information that is not currently declared in your Funding Statement. However, funding information should not appear in the Acknowledgments section or other areas of your manuscript. We will only publish funding information present in the Funding Statement section of the online submission form.

Please remove any funding-related text from the manuscript and let us know how you would like to update your Funding Statement. Currently, your Funding Statement reads as follows:

This study was partially supported by the Hunter Medical Research Institute/Greaves Family Postgraduate Top-Up Scholarship (Grant number G1701582). Wollega University (first author's employer organization) facilitated data collection process. The funders had no role in study design, data collection and analysis, decision to publish, or preparation of the manuscript.

Response: The funding statement has been updated as follows.

This study was partially supported by the Hunter Medical Research Institute/Greaves Family Postgraduate Top-Up Scholarship (Grant number G1701582). Wollega University (first author's employer organization) facilitated the data collection process. TRF is supported by The University of Newcastle International Postgraduate Research Scholarship (UNIPRS) and The University of Newcastle Research Scholarship Central 50:50 (UNRSC 50:50). Dr Melissa Harris is supported by an Australian Research Council Discovery Early Career Researcher Award (DECRA). The funders had no role in study design, data collection and analysis, decision to publish, or preparation of the manuscript.

# **Reviewer#1**

Reviewer #1: This paper describes a cross-sectional study examining contraceptive use among women living with HIV in Ethiopia. The authors sought to describe types of contraceptives used, prevalence of unmet need, and predictors of contraceptive use among a sexually active women who were not currently pregnant or planning to become pregnant. They also employed the Health Believe Model to assess beliefs about facilitators and barriers to contraceptive use and how this was associated with use. The authors found that contraceptive use was high, and identified a number of factors associated with contraceptive use, including marital status, number of children born since HIV diagnosis, and perceived susceptibility to unintended pregnancy. The authors conclude that their findings highlight the need for strengthening family planning services.

This research addresses an important topic, and while none of the findings are surprising the data collection appears to be well conducted and the findings can contribute to a body of literature assessing unmet need. However, as written, I have major concerns with this manuscript that I would recommend addressing before publication, as well as a number of minor comments.

**Major concerns:**

**Comment:** 1. There were a number of grammatical errors, unclear sentences, and paragraphs that mixed concepts. The manuscript is also much lengthier than necessary, and does not focus very well on the topic of interest. I have noted some of these below, but it needs more editing than what is listed below. While I do think the science is sound, I would not recommend publication until there is thorough editing for grammar, sentence structure, and flow.

**Response:** The manuscript has been revised for grammar, sentence structure and flow. We have also improved the focus of the paper.

2. It is unclear why stepwise selection was used “to control for potential confounders” when this appears to be an exploratory analysis to identify factors independently associated with contraceptive use. Stepwise regression is often used when you have a specific exposure and outcome of interest, and you want to identify factors that may be confounding the relationship between those two variables. Here, you did not identify a specific exposure of interest, but still used this technique to identify a final model which you then presented as adjusted ORs, but then also presented those that didn’t make it into the model as unadjusted ORs. It wasn’t really addressed what it means for something to be in the final model or not. I believe a more appropriate approach is to present the findings of the multivariate model without stepwise selection. Those that are significant at your pre-specified alpha level are then the variables that are independently associated with contraceptive use when controlling for the other variables in your model. I think this analysis should either be changed, or better explained how you interpret your results.

Response: We have revised the methods of analyses and clarified the analysis approach in the methods section of the manuscript. In the revised approach, we initially used log-binomial regression models to obtain the prevalence ratio (Deddens and Petersen 2008, Williamson, Eliasziw et al. 2013), although convergence was not achieved, even when using the COPY adjustment method. As a result, Poisson regression models with robust standard errors were used to estimate prevalence ratios (Deddens and Petersen 2008, Williamson, Eliasziw et al. 2013). Poisson regression models were initially used to identify potential factors associated with contraceptive use. Variables that were considered potential independent risk factors from the univariate analyses (p-value<0.2) were considered for a final multivariate model, to control for confounding. Collinearity was evaluated for the adjusted model (results not shown). The strength of the association between the outcome variable and independent variables was expressed in prevalence ratio (PR) with a 95% confidence interval. Our interpretations have been revised based on the prevalence ratio.

3. I couldn’t really identify how the authors defined unmet need. This is simple to include and is necessary to compare to other studies that are assessing unmet need.

Response: We have included the following definition of unmet need for contraception in the introduction. The definition now reads:

According to the World Health Organization (WHO) definition, women with unmet need are “those who are fecund and sexually active but are not using any method of contraception, and report not wanting any more children or wanting to delay the next child” (WHO).

**Minor concerns:**

**Introduction**
1. This section in general is lengthier than it needs to be.

Response: We have edited and shortened the introduction section.

2. The first paragraph has a number of sentences with unclear wording or grammatical errors. This includes lines 48-49 “The 2015 WHO guideline avoids medical or clinical barriers”, line 50 “Along this improvement”, and line 55 “pregnancies has also benefits”.

Response: We have made amendments to the first paragraph of the introduction. The statements now read:

The 2015 WHO guideline states there should be no criterion barrier in the initiation of ART which will help in reductions in HIV-related morbidity and mortality.

With the current ART expansion, meeting contraceptive needs is crucial to not only achieving fertility goals but also to contributing to reductions in maternal morbidity and mortality, child mortality and children who being orphaned.

The statement containing, ‘“...pregnancies has also benefits” has been deleted.

3. Line 60-61 “Due to ART [16, 17], WLHIV need clear reproductive life plans with ready access to contraception” – What does this mean? Because they are living longer?

Response: The statement has been amended and now reads, “With improved survival of HIV positive population because of ART, WLHIV need clear reproductive life plans with ready access to contraception.”

4. Line 71: I believe this should say “prevention of both pregnancy and HIV transmission”.

Response: The statement has been amended and now reads, “While it has been advised by the WHO that WLHIV should choose any contraceptive methods similar to HIV-negative women, the contraceptive choice in the presence of HIV appears more complex because WLHIV are required to balance the prevention of both pregnancy and HIV transmission.”

5. Line 71-74: Most of these things would also be a concern for women without HIV- should focus on transmission.

Response: The statements have been amended and focused on both HIV (resistant strain) and STIs transmissions. The statement now reads:

WLHIV are required to consider a risk of transmission of HIV/sexually transmitted infections (STI) to a partner as well as the risk of acquiring STIs/different HIV strains (Berhan 2008, Gruskin, Firestone et al. 2008).

6. Line 88: should say “which include”

Response: The statement now reads, ‘Cues to action are internal and external signals used to activate readiness to change’.

**Methods**
7. Lines 100-104: A lot of this detail, like exact population size and distance to the capital, is unnecessary

Response: We have deleted information regarding exact population size and distance to the capital.

8. Lines 109: Should say “fecundity” or “being fecund”.

Response: The statement now reads as ‘being fecund’.

9. Line 111-112: You say an inclusion criterion includes having a demand of contraception. How is this defined? Did they report this, or was it determined based on meeting eligibility criteria for a-c?

Response: The statements now reads as ‘sexually active HIV-positive women who met the eligibility criteria and were included for analysis’.

10. For a number of variables, including length of time the women were using the method(s) without interruption and time on ART in years, you have some categories that are overlapping (e.g. >12-36, ≥36).

Response: The categories have been amended so that contraceptive continuation was determined by the length of time the women were using the method(s) without interruption (≤12 months, >12 to < 36 months, and ≥36 months).

11. Lines 176-177: I think the caps here are unnecessary. You use italics for emphasis elsewhere; I think you could use that here as well.

Response: The capitalisation of words has been removed from this sentence. It now reads, ‘pregnancy intention at conception of all live births in the last five years of these women was categorized into intended (*wanted then*), mistimed (*wanted later*) and unwanted (*not at all*).’

12. Lines 199-206: I’m a little confused about this. Was this done for a previous analysis or specifically for this analysis? If done for a previous analysis, I would just state that summary scores were derived using PCA and the methods have been previously described, then cite where this has happened. If this was done specifically for this analysis, I would move it to the statistical analysis section.

Response: The PCA was carried out for this study and it has been moved to the statistical analysis section.

13. Lines 211 and 214: p-value shouldn’t be capitalized

Response: The p-value is no longer capitalized in the manuscript.

14. Statistical analysis section: Poisson with robust standard errors to get prevalence ratios performs better than logistic regression for ORs with cross-sectional data.

Response: Prevalence ratios are now presented using Poisson regression models with robust standard errors (Deddens and Petersen 2008, Williamson, Eliasziw et al. 2013) (see response to major comment #2 for details).

**Results**
15. Line 223: This is the first time unmet need is introduced and it was not defined.

Response: The definition of unmet need for contraception has been included in the introduction (please see response under major comment #3).

16. Table 1: This is stylistic, but it is easier for the eye if the n and % are near each other (so n is right aligned and % is left aligned)

Response: The table has been amended so that the number and percent are closer to each other.

17. Table 1: The first foot note doesn’t seem to actually appear in the table? Also footnotes should read left to right, top to bottom, so the other footnote should be switched.

Response: As per the suggestion, we have amended the footnotes.

18. Line 239: Should be “reporting”

Response: The statement now reads, ‘Results in Table 2 show the HIV-related, reproductive-related and HBM factors, with 111 (30.9%) women reporting that they learned of their HIV status 10 or more years ago.’

19. Line 248: You should restate what susceptibility and severity are referring to

Response: We have restated that susceptibility referred to pregnancy (unintended) and perceived severity referred to being pregnant while living with HIV. The statement now reads, ‘The percentage of women who reported high perceived susceptibility to pregnancy (unintended) and severity of being pregnant while living with HIV were 20.8% and 30.7%, respectively.’

20. Lines 270-282: Referring back to my comment above about your statistical approach, this way of reporting is a bit confusing. If the ones you report as unadjusted did not remain significant in the final model, this should be stated.

Response: We have changed the methods of analysis (please see the response #2 under major comment and #14 under minor comment). Robust Poisson regression was used and all variables that have an association with contraceptive use have been included in the multivariate model. In addition, we have modified the interpretations of findings regarding the predictors. The method of interpretation is now changed to ‘risk’ and is focused on the adjusted model (Deddens and Petersen 2008, Williamson, Eliasziw et al. 2013). We have deleted the details of reports of unadjusted model (lines 270-282).

21. Lines 285-299: This final section is descriptive and would make more sense to be presented earlier.
Response: We have moved the description of contraceptive use characteristics before the predictors.

**Discussion**
22. Lines 314-315: How does the prevalence of unmet need found in your study compare to that found in other studies?

Response: Unmet need for contraception has been compared with another similar study. It now reads:

A cross-sectional study at Saint Paul’s Hospital Millennium Medical College, Addis Ababa, Ethiopia also showed a similar magnitude of unmet needs for contraception among married WLHIV (25.1%) (Abubeker, Fanta et al. 2019).

23. Line 333-335 “There is considerable evidence that utilizing pre-exposure prophylaxis (although not currently available in Ethiopia) could address some of the gaps related to HIV transmission among non-condom users [40]” This does not seem particularly relevant considering your population is living with HIV. Since this paper is very lengthy it would be better to stick with things relevant to your findings.

Response: The statement has been deleted as per the suggestion.

24. Line 361: This is repetitive

Response: We have deleted that statement.

25. Line 362-364: Wording unclear

Response: We have amended the statement that the majority of contraceptive users reported that they obtained their contraceptive methods from health centers. It now reads:

Given health centers are available closer to the community (distance), WLHIV might be more likely to obtain contraception from the health centers as hospitals are usually further away (Shiferaw, Spigt et al. 2017).

26. Line 382-383 “were about fourteen times more likely to use contraception compared to women who never had a child after HIV diagnosis” You should acknowledge here that you had an extremely small sample size and wide confidence intervals for this finding.

Response: Methods of analysis has been changed and it is now reported as prevalence ratio. It now reads:

The prevalence of contraceptive use among WLHIV who had two or more children since HIV diagnosis was 1.31 times higher than WLHIV who never had a child after HIV diagnosis.

27. Lines 383-388: It seems like this would be the expected outcome, as women with more children are probably less likely to want more children and are likely older. Did you consider this, or look at this at all in your data? Seems like a more simple explanation than the ones listed here.

Response: Both number of children living at home and number children born after HIV-diagnosis have been adjusted in the multivariate model but the number of children born after HIV-diagnosis remained significantly associated with contraceptive use. Age was checked as a possible factor, but there was little evidence to support it in the univariate and multivariate analyses. Further investigation using a larger study may be able to better examine the impact of number of children and age on contraceptive practice.

28. Line 391: “This perception evaluated the chance of unintended pregnancies.” It’s not clear to me what this means.

Response: The statement now reads, ‘The perceived susceptibilities assessed the perceived chance of pregnancy (unintended) if contraception is not used.’

29. Line 396: It’s unclear to me what “ruled out” means in this context.

Response: The statement has been amended to clarify that the effect of perceived benefits cannot be excluded. It now reads:

Despite only showing an association in an unadjusted model, the effect of perceived benefits cannot be excluded.

30. Line 398: I think the “perceived susceptibility” here should be perceived benefit?

Response: We have amended the statement.

Women with a high perception about the benefits of contraceptive use had an increased risk of contraceptive use compared to women with a low perceived benefits.

31. Lines 399-402: “Essentially, improved perceptions regarding contraception such as long-term protection, convenience of methods, effectiveness as well as dual protection for these women are critical factors identified by the HBM in our study and might increase uptake of contraception.” Did your findings really support this? How do you interpret the finding that most of your “HBM factors” did not appear in your final model?

Response: Given there were 30 questions about perceptions, the summed score was used. Our finding is based on cumulative score (the PCA summed score). The statement has now been amended to reflect the effects of contraceptive counselling regarding the perceptions and further supported by references. It now reads:

However, contraceptive counselling regarding long-term protection of contraception, convenience of methods, effectiveness, as well as dual protection might increase adherence (Halpern, Lopez et al. 2013) and uptake of contraceptive use (Dehlendorf, Krajewski et al. 2014).

# Reviewer #2

Title: Contraceptive use among sexually active women living with HIV in western Ethiopia
Reviewer’s report:

Thank you for the opportunity to review the manuscript entitled “Contraceptive use among sexually active women living with HIV in western Ethiopia by Feyissa et al. on a very important subject. This is a well-written manuscript that is grounded in the Health Belief Model with clear objective and well written introduction. The methods are sound. The results are adequately presented and well synthesized against previous evidence and the model adapted as the conceptual framework. The authors also acknowledged the weaknesses/limitations in their study. Their conclusions are consistent with the evidence and arguments presented and addressed the main question posed. I strongly believe this manuscript makes a meaningful contribution to the field of public health and therefore, should be considered for publication pending editor's decision.

Nonetheless, these are few suggestions, which I think could strengthen the paper before publication.

**Abstract**
1. This is a well written abstract. However the authors can add a brief background to the abstract.

Response: We have included a brief background statement to the abstract.

Contraception can help to meet family planning goals for women living with HIV (WLHIV) as well as support the prevention of mother to child transmission of HIV (PMTCT). However, there is little research into the contraceptive practice among sexually active WLHIV in Ethiopia.

2. Line 21 Please specify the exact date (e.g 1st March to 1st June 2018)

Response: The statement now reads, ‘…conducted from 19^th^ March to 22^nd^ June 2018 in western Ethiopia’.

3. Line 29 should read… were associated with increased “odds of” contraceptive practice.

Response: Given we have changed the method of analysis to robust Poisson regression, we have amended and now reads, ‘increased risk of’.

4. Line 41, keywords: Health belief should be “health belief model”

Response: We have amended and now it reads, ‘health belief model’.

**Introduction**
5. Very good introduction with adequate review of literature. However, this can be more strengthened when discussed within the context of the SDGs. Specifically goal 3.3

Response: We have included a statement regarding the importance to SGDs 3.3 of ending the epidemics of AIDS by 2030. The addition to the introduction regarding SGDs 3.3 reads:

Therefore, strengthening family planning programs could play an important role in ending the epidemics of AIDS by 2030 (the Sustainable Development Goal 3.3) (Lim, Allen et al. 2016).

6. Line 63-64, can you please specify some of these studies?

Response: The statement has supporting references now.

**Conceptual framework**

7. Please this is a very minor comment. I would suggest the authors bring a sub-heading “conceptual framework” and also provide a diagrammatical representation of the variables considered in the model and how they are related to the outcome variable (s).

Response: Reviewer #1 suggested that the manuscript is too lengthy, so we are cognisant of balancing the need to further information against concise text and word length. In an effort to achieve this, the conceptual framework has been included as figure (see Fig.1). The relationship of the HBM factors with outcome variable has been shown in the figure. Furthermore, it has been indicated in the methods sections that personal perceptions related to conception and contraception amongst WLHIV was measured using 30 questions as guided by existing HBM research on contraceptive use (Brown, Ottney et al. 2011, Hall 2012).

**Materials and Methods**

8. Line 98 should be read “Materials and Methods”

Response: As per the suggestion, we have amended and now reads, ‘Materials and methods’.

9. Is it possible to provide a map indicating the study settings?

Response: Reviewer #1 suggested that details such exact population size and distance to the capital are unnecessary. As such, we have not included a map.

10. Can you provide a sample of the instrument used for the data collection as an attachment (supplementary file)?

Response: The questions were adapted from Demographic and Health Survey (<https://dhsprogram.com/pubs/pdf/FR328/FR328.pdf>), and Family Planning 2020 questionnaires (<https://www.familyplanning2020.org/>). Links to these surveys have been added to the manuscript as we do not own the copyright.

11. Line 101 can you please specify the exact date?

Response: The exact date has been included (19^th^ March to 22^nd^ June 2018).

12. From Figure 1, the total women surveyed are 1082. Was this the total number of HIV positive women attending HIV clinics across the 4 facilities you were able to reach? If yes, how many were in each facility? How did you ensure representativeness across the facilities?

Response: We have included details regarding the sampling procedure. A total of 2,445 reproductive-age WLHIV attended the four facilities at the time of data collection. The study participants were recruited using a systematic random sampling of HIV-positive women attending HIV clinics across four health facilities (two hospitals and two health centers).

‘The recent treat‐all policy meant there was no criteria barrier to starting ART (World Health Organization 2015), encouraging all PLHIV to commence ART immediately after HIV-positive diagnosis. To facilitate this in Ethiopia, a unique ART number is assigned to PLHIV to monitor their ART treatment and HIV management, and to coordinate care across different settings. For the recruitment process, a list of ART clients (each with a unique ART number) was obtained from the daily appointment calendar, excluding the personal identifiers of each client. Among 2,445 reproductive-age HIV-positive women, a systematic random sampling of every second woman from each health facility was used to select study participants with selected WLHIV invited to participate in the study. All participants were contacted at a HIV clinic of the selected health institutions when they came for their ART services.’

13. Although you have given a very nice flow of the way the 360 sample was arrived at but kindly explain how the systematic random sampling was used to arrive at the unit of analysis.

Response: As indicated in schematic presentations, the total participants fulfilling the criteria were included in the analysis (360 sexually active women living with HIV). A total number of WLHIV surveyed were 1082. We have amended the section on the sampling procedure to demonstrate how we used a systematic random sampling technique.

As shown in Fig 2, among the 1,082 women who were surveyed, 360 sexually active HIV-positive women who met the eligibility criteria were included for analysis (see response to number #12 for details regarding systematic random sampling technique).

14. Line 119, please where was the piloting done? And what informed the choice of that place for the piloting? How many WLHIV were used for the piloting?

Response: Details of pilot testing has been included.

Because of ethical and logistic reasons, a pilot study was carried out among 30 participants in the health facilities that were selected for main data collection. These women were not included the main data collection.

15. Line 122, please how long were the nurses trained and on what?

Response: We have amended the text to indicate that the nurses’ training and pilot testing was conducted for five days on the questionnaire, consenting procedures, and completing the survey. The statements now read:

Data quality was maintained by training data collectors on the questionnaire, consenting procedures and completing surveys.

The training and pilot testing were conducted for five days.

16. Line 169-170, please how was data on CD4 counts collected, self-reported?? or from their records?? This should be acknowledged at the discussion section?

Response: We have indicated in the limitations section that all data were based on self-report. We have also indicated in the methods section that the data collection was conducted face-to-face with mobile-based survey.

17. Line 203-205, please what informed the categorization of the “susceptibility” into tertiles (example: high susceptibility, medium susceptibility and low susceptibility?

Response: We have supported the statements that the results of PCA can be categorized into tertiles.

**18. Statistical analyses.**

a. Please how was the adjustment done? (see Table 3).

Response: The final multivariate model was determined by including variables that were significantly independently associated with contraceptive use.

b. Please what informed the choice of the reference categories?

Response: Reference categories were selected based on previous studies and what made sense.

c. Kindly specify how the missing values were treated at the inferential analysis stage.

Response: The missing values have excluded in robust Poisson model. Four observations were excluded from the final model due to missing values.

Results
19. I suggest you bring the Table 4 (Selected contraceptive indicators among sexually active women with HIV in western Ethiopia, 2018) before Table 3 (Factors associated with contraceptive practice among sexually 283 active women living with HIV in western Ethiopia, 2018) to make all the descriptive results come before the inferential results (logistic regression).

Response: The results section has been modified so that the descriptive results come first.

**Discussion**
20. Line 304, the sentence… “supporting the PMTCT programs by preventing unintended pregnancies” should be supported with evidence

Response: This statement has been supported by a reference.

21. The authors have done an excellent job by acknowledging the weaknesses of their study including the study design, however, they did not acknowledge the strength of their study. I also suggest this should be given a sub-heading for easy reading.

Response: We have now included strength of the study that the study applied the health belief model and examined contraceptive use among sexually active WLHIV. ‘Strengths and limitations’ of the study has now been included as sub-heading.

**Conclusion**
22. Well written conclusions emanating from the study’s findings. Despite this can you please bring a subheading “conclusions and policy implications” directly after the strength and weaknesses of the study to make it easy for readers who wish to go to the conclusion section directly do that at ease.

Response: ‘Conclusion’ heading (although optional) has been included as per the Plos One guideline.

# **References**

Abubeker, F. A., M. B. Fanta and V. K. Dalton (2019). "Unmet Need for Contraception among HIV-Positive Women Attending HIV Care and Treatment Service at Saint Paul’s Hospital Millennium Medical College, Addis Ababa, Ethiopia." International journal of reproductive medicine **2019**.

Berhan, Y. (2008). "Attitude of people living with HIV/AIDS to be a biological parent and their knowledge on MTCT reduction methods." Ethiop Med J **46**(4): 335-347.

Brown, W., A. Ottney and S. Nguyen (2011). "Breaking the barrier: the Health Belief Model and patient perceptions regarding contraception." Contraception **83**(5): 453-458.

Cahill, N., E. Sonneveldt, J. Stover, M. Weinberger, J. Williamson, C. Wei, W. Brown and L. Alkema (2018). "Modern contraceptive use, unmet need, and demand satisfied among women of reproductive age who are married or in a union in the focus countries of the Family Planning 2020 initiative: a systematic analysis using the Family Planning Estimation Tool." The Lancet **391**(10123): 870-882.

Central Statistical Agency (CSA) [Ethiopia] and ICF (2016). Ethiopia Demographic and Health Survey 2016. Addis Ababa, Ethiopia, and Rockville, Maryland, USA.

Deddens, J. and M. Petersen (2008). "Approaches for estimating prevalence ratios." Occupational environmental medicine **65**(7): 501-506.

Dehlendorf, C., C. Krajewski and S. Borrero (2014). "Contraceptive counseling: best practices to ensure quality communication and enable effective contraceptive use." Clin Obstet Gynecol **57**(4): 659-673.

FDRE Ministry of Science and Technolgy (2014). Ethiopian National Ethics Guideline. Addis Ababa, Ethiopia, FDRE Ministry of Science and Teachnolgy,. **2018**.

Gruskin, S., R. Firestone, S. MacCarthy and L. Ferguson (2008). "HIV and Pregnancy Intentions: Do Services Adequately Respond to Women’s Needs?" American Journal of Public Health **98**(10): 1746-1750.

Hall, K. S. (2012). "The Health Belief Model can guide modern contraceptive behavior research and practice." J Midwifery Womens Health **57**(1): 74-81.

Halpern, V., L. M. Lopez, D. A. Grimes, L. L. Stockton and M. F. Gallo (2013). "Strategies to improve adherence and acceptability of hormonal methods of contraception." Cochrane database of systematic reviews(10).

Heale, R. and A. Twycross (2015). "Validity and reliability in quantitative studies." Evidence-based nursing **18**(3): 66-67.

Lim, S. S., K. Allen, Z. A. Bhutta, L. Dandona, M. H. Forouzanfar, N. Fullman, P. W. Gething, E. M. Goldberg, S. I. Hay and M. Holmberg (2016). "Measuring the health-related Sustainable Development Goals in 188 countries: a baseline analysis from the Global Burden of Disease Study 2015." The Lancet **388**(10053): 1813-1850.

Shiferaw, S., M. Spigt, A. Seme, A. Amogne, S. Skrøvseth, S. Desta, S. Radloff, A. Tsui and D. GeertJan (2017). "Does proximity of women to facilities with better choice of contraceptives affect their contraceptive utilization in rural Ethiopia?" PloS one **12**(11): e0187311.

WHO. "Unmet need for family planning." 2020, from <https://www.who.int/reproductivehealth/topics/family_planning/unmet_need_fp/en/>.

Williamson, T., M. Eliasziw and G. H. Fick (2013). "Log-binomial models: exploring failed convergence." Emerg Themes Epidemiol **10**(1): 14.

World Health Organization (2015). GUIDELINE ON WHEN TO START ANTIRETROVIRAL THERAPY AND ON PRE-EXPOSURE PROPHYLAXIS FOR HIV. Geneve, WHO.
